# CircInpp5b Ameliorates Renal Interstitial Fibrosis by Promoting the Lysosomal Degradation of DDX1

**DOI:** 10.3390/biom14060613

**Published:** 2024-05-23

**Authors:** Xi Fang, Chengyuan Tang, Dong Zeng, Yi Shan, Qianfang Liu, Xuemin Yin, Ying Li

**Affiliations:** 1 Department of Nephrology, The Second Xiangya Hospital, Central South University, Changsha 410011, China; fangxi0321@csu.edu.cn (X.F.); tangchengyuan@csu.edu.cn (C.T.); zengdongdoc@163.com (D.Z.); sy313131sy@163.com (Y.S.); shiguanglqf@163.com (Q.L.); yinxuemin0104@163.com (X.Y.); 2 Key Laboratory of Kidney Disease and Blood Purification in Hunan Province, Changsha 410011, China

**Keywords:** circRNA, circInpp5b, renal interstitial fibrosis, DDX1, lysosomal degradation

## Abstract

Renal interstitial fibrosis (RIF) is a classic pathophysiological process of chronic kidney disease (CKD). However, the mechanisms underlying RIF remain unclear. The present study found that a novel circular RNA, cirInpp5b, might be involved in RIF by high-throughput sequencing. Subsequent experiments revealed that circInpp5b was reduced in UUO mouse kidney tissues and TGF-β1-treated proximal tubular cells. The overexpression of circInpp5b inhibited RIF in UUO mice and prevented extracellular matrix (ECM) deposition in TGF-β1-treated proximal tubular cells. Furthermore, overexpression of circInpp5b down-regulated the protein level of DDX1. Mechanistically, circInpp5b bound to the DDX1 protein and promoted its lysosomal degradation. Collectively, the findings of our study demonstrate that circInpp5b ameliorates RIF by binding to the DDX1 protein and promoting its lysosomal degradation.

## 1. Introduction

Chronic kidney disease (CKD) is currently a health problem worldwide [1]. CKD affects about 10% of the world’s population and eventually leads to end-stage renal disease (ESRD). ESRD frequently necessitates renal replacement therapy, which brings a huge burden to the global medical system [2]. Renal interstitial fibrosis (RIF) is a classic pathophysiological process of CKD progressing to end-stage renal failure [3]. RIF is characterized by damage, cell activation, and extracellular matrix (ECM) deposition in the kidneys. The occurrence and development of RIF involve the activation of key cellular or signaling pathways including tubular epithelial cells, TGF-β1, Notch, etc. The epithelial cells secrete ECM-related proteins. Excessive fibrotic matrix is deposited during the repair process after interstitial injury [4,5]. However, the mechanism underlying RIF remains unclear.

Circular RNAs (circRNAs) are characterized by a closed circular structure without a poly-adenylated tail [6]. In 1976, Sanger discovered an RNA in viroids and described it for the first time as a single-stranded RNA molecule with a closed circular structure [7]. Since the early 21st century, fueled by RNA sequencing technology, studies on circRNAs have shown explosive growth [8,9,10,11,12]. Studies showed that circRNAs interacted with miRNAs and participated in the translation of target genes [13,14]. Moreover, circRNAs could also combine with proteins and function as a sponge, scaffold, etc. In particular, circRNAs interacted with proteins and participated in their transcription, translation, and post-translational modification [8,15,16,17,18]. Previous studies showed that circRNAs participated in kidney diseases such as kidney tumors, acute kidney injury, and diabetic nephropathy [19]. More importantly, there have been many studies in renal fibrosis. For example, there were several studies about circHIPK, circRNA_30032, circRNA_33702, and circular RNA ACTR2 in RIF [20,21,22,23,24]. However, more studies revealed that circRNAs functioned as miRNA sponges, and fewer studies proved that circRNAs interacted with proteins. Thus, the mechanism underlying circRNAs in RIF remains unclear. For further investigation, our study found that a novel circRNA, named circInpp5b, might be involved in RIF by high-throughput sequencing, and this circRNA has not been studied yet. Through using the RPISeq database for predicting interaction between RNAs and proteins, we discovered that circInpp5b might bind to the DDX1 protein.

The DEAD-box RNA (DDX) helicase family has a characteristic motif, Asp-Glu-Ala-Asp (DEAD), with domains of extremely high conservation, regulating RNA helicase activity, ATPase activity, and substrate binding [25]. DDX helicases also participated in RNA metabolism [26]. DDX1, the earliest found DDX helicase, is an RNA-binding protein [27,28]. One study showed that DDX1 promoted the maturation of precursor miRNAs and participated in tumors [29]. Another study showed that circLONP2 regulated the maturation of miRNA by regulating DDX1 in colon cancer, which suggested DDX1 might bind to circRNAs [30]. At present, DDX1 is rarely studied in kidney diseases. Therefore, further research is needed to investigate the specific effect of DDX1 in RIF.

In this study, we identified a novel circRNA, named circInpp5b, that might play a beneficial role in RIF. Further investigations revealed that circInpp5b bound to the DDX1 protein, promoted its lysosomal degradation, and, finally, ameliorated RIF, which suggested that circInpp5b might be a novel therapeutic target for RIF.

## 2. Materials and Methods

### 2.1. Animal Models

Male C57BL/6 mice (20–25 g weight) aged 8–12 weeks old, bought from Slack Jingda laboratory animal company (Hunan, China), were raised in Second Xiangya Hospital. Food and water were freely available every day. The animal models we used comprised unilateral ureteral obstruction (UUO) mice. The mice were all routinely given intraperitoneal anesthesia by using pentobarbital. After skin preparation, the left kidney was found. Then, we continued to look for the ureter at the lower pole of the kidney. After ligating both ends of the ureter, the middle of the ureter was cut, then the kidney and ureter were equalized. For the Sham group, we only separated the ureter and did not ligate it. After surgery for 3, 7, and 14 days, the kidney tissues and blood samples were collected. The animal experiments were approved by the Institutional Animal Care and Use Committee (IACUC) at the Second Xiangya Hospital of Central South University (No. 20230920).

### 2.2. Lentivirus Intrarenal Injections

The construction of circInpp5b or DDX1 lentivirus and scramble was carried out by genechem (Shanghai, China). In this study, the concentration of lentivirus was 3.5 × 10^8^ TU/mL, 100 uL per mouse. The mice were all routinely given intraperitoneal anesthesia by using pentobarbital. After skin preparation, the kidney and surrounding adipose tissue were exposed. Then, we continued to look for the renal pedicle and used forceps to block it reversibly. The lentivirus or scramble was extracted with a 25–G needle and inserted very slowly into the renal parenchyma from the lower part of the left kidney. Four sites were selected for injection, and the needle was used for 10 s each time. Then, we released the renal pedicle. We were careful when putting the kidney in place.

### 2.3. Cell Culture and Treatment

The renal tubular cell, BUMPT cell line, was gifted by Drs. William Lieberthal and John Shwartz at Boston University. The cells were cultured in DMEM (Gibco, Waltham, MA, USA), 10% FBSBiological Industries, Cromwell, CT, United States, and antibiotics. The culture incubator had a temperature of 37 °C and a 5% CO_2_ atmosphere. For certain experiments, 293T cells were used. The same culture media and environment were applied to the 293T cells. In this study, the vitro model of RIF comprised TGF-β1-treated BUMPT cells. When the cell density was 30–40%, we changed the serum-free medium overnight; then, TGF-β1 (Sino Biological, Beijing, China, #10804-HNAC, 5 ng/mL, 24 h) was added. Other treatments used in this study include the following: MG132 (MCE, Shanghai, China, #HY-13259, 5 µM, 6 h), chloroquine (MCE, #HY-17589A, 20 µM, 24 h), and NH_4_Cl (SigmaAldrich, Missouri, MO, USA, #213330, 20 mM, 24 h).

### 2.4. Cell Transfection

The construction of circInpp5b and Ddx1 plasmid or siRNA was carried out by genechem (Shanghai, China). When cell density was 50–70%, transfection was performed according to the instructions of lipo2000 (Invitrogen, Carlsbad, CA, USA). We changed with serum-free non-antibiotics culture medium for 6–8 h and then changed with complete medium. The cells were cultured, and the following interventions were performed when they reached the corresponding density.

### 2.5. Immunohistochemistry (IHC) Staining

The paraffin sections were deparaffinized. Next, 0.1 M sodium citrate was added for antigen repair. Then, endogenous peroxidase blockers and goat serum were added. Primary antibodies, including Col-I (Affinity Biosciences, Cincinnati, OH, USA, #AF7001, 1/200), Fn (Abcam, Cambridge, United Kingdom, #ab2413, 1/400), and DDX1 (Proteintech, Wuhan, China, #11357-1-AP,1/200), were added overnight. Serum was used as a negative control instead of a primary antibody. A secondary antibody (Zsbio, Beijing, China, PV-9005) was added. DAB solution was applied for 1–2 min accordingly. Hematoxylin was used for 8 s. The sections were dehydrated and transparent, and they were observed under a light microscope (Leica DMI 3000 B, Leica, Wetzlar, Germany). Protein deposition was measured by area (%). Image-Pro Plus 6.0 was used to analyze images of at least 10 fields per mouse and 4 mice for each group.

### 2.6. Hematoxylin–Eosin (HE) Staining

The paraffin sections were deparaffinized, and hematoxylin and eosin solution were added. After washing, the sections were dehydrated and transparent, and they were observed under a light microscope. Tubule atrophy was scored by area (%): 0: no damage, 1: <25%, 2: 25–50%, 3: 50–75%, and 4: >75%. Images were analyzed, at least 10 fields per mouse and 4 mice for each group, by 2 scientists.

### 2.7. Masson’s Trichrome Staining

The paraffin sections were deparaffinized and immersed in hematoxylin solution, phosphomolybdic acid solution, and aniline blue solution. The sections were dehydrated and transparent, and they were observed under a light microscope. Collagen deposition was measured by area (%). Image-Pro Plus 6.0 was used to analyze images of at least 10 fields per mouse and 4 mice for each group.

### 2.8. Periodic Acid-Schiff (PAS) Staining

Similar to HE staining, the paraffin sections were deparaffinized and supplemented with periodic acid and Schiff reagent. The sections were dehydrated and transparent, and they were observed under a light microscope.

### 2.9. Fluorescence In Situ Hybridization (FISH)

The circInpp5b probes were synthesized by GenePharma (Shanghai, China). FISH assays were also performed by using kits from GenePharma (Shanghai, China). In brief, 4% paraformaldehyde was added for 15 min. After being permeabilized, the cells were incubated with a probe mixture (GenePharma, Shanghai, China, 1 µM) at 37 °C for hybridization overnight. DAPI solution was added for 20 min in the dark. The cells were evaluated by using a fluorescence microscope (Leica DMI 3000 B, Leica, Wetzlar, Germany). The paraffin sections were deparaffinized and hydrated. Then, proteinase K was added. The probe mixture (GenePharma, 1 µM) was added to hybridization at 37 °C overnight. DAPI solution was added for 20 min in the dark. The sections were evaluated by using a fluorescence microscope (Leica DMI 3000 B, Leica, Wetzlar, Germany).

### 2.10. Western Blot

Firstly, RIPA buffer and Protease Inhibitor were added to lyse the kidney tissues and cells. Next, 8% SDS-PAGE gel was used to separate the proteins. After electrophoresis, the proteins were transferred to the PVDF membranes. The membranes were blocked by 5% milk for 60 min. And primary antibodies, including Col-I (Abcam, #ab260043, 1/1000), Fn (Abcam, #ab2413, 1/1500), and DDX1 (Proteintech, #11357-1-AP,1/1000), were added overnight at 4 °C. A secondary antibody (Proteintech, #SA00001-2, 1/4000) was added for 1 h. Finally, a chemiluminescent substrate (Thermo Scientific, Waltham, MA, USA) was used to visualize target proteins.

### 2.11. Quantitative Real-Time PCR (qRT-PCR)

Trizol reagent (AG21102) was added to extract total RNAs. After cleaning the gDNA, the cDNA was obtained from RNA reverse transcription by using a kit (AG11728), and equal amounts of cDNA were subjected to quantitative real-time PCR, carried out by using a kit (AG11701). The circRNA and mRNA levels were normalized by β-actin. The primers are indicated in Table 1.

### 2.12. RNA Immunoprecipitation (RIP) 

FLAG-Ddx1 plasmid was transfected into 293T cells, and the subsequent steps were based on the RIP kit (BersinBio, Guangzhou, China, #Bes5101). Firstly, the cells were lysed. Then, according to IP, IgG, and Input, we divided the cell lysate samples into three parts. Next, 5 ug each of the following experimental antibodies were added to the IP and IgG samples: Flag (Sigma-Aldrich, #F1804) and Mouse IgG (Proteintech, #B900620). The IP and IgG samples with antibodies were incubated in a vertical mixer (10 rpm) at 4 °C for 16 h, then added with Protein A/G beads and incubated in a vertical mixer (10 rpm) at 4 °C for 1 h. The beads were collected using a magnetic rack. Polysome elution buffer was added, and the RNAs were extracted using trizol reagent. The subsequent steps were the same as for the qRT-PCR.

### 2.13. Statistical Analysis

Means ± standard deviation (SD) is used to indicate the values of results. Statistical analysis was performed using GraphPad Prism Version 7.0.

## 3. Results

### 3.1. CircInpp5b Is Down-Regulated in UUO Mice and BUMPT Cells Treated with TGF-β1

**Figure 1:**
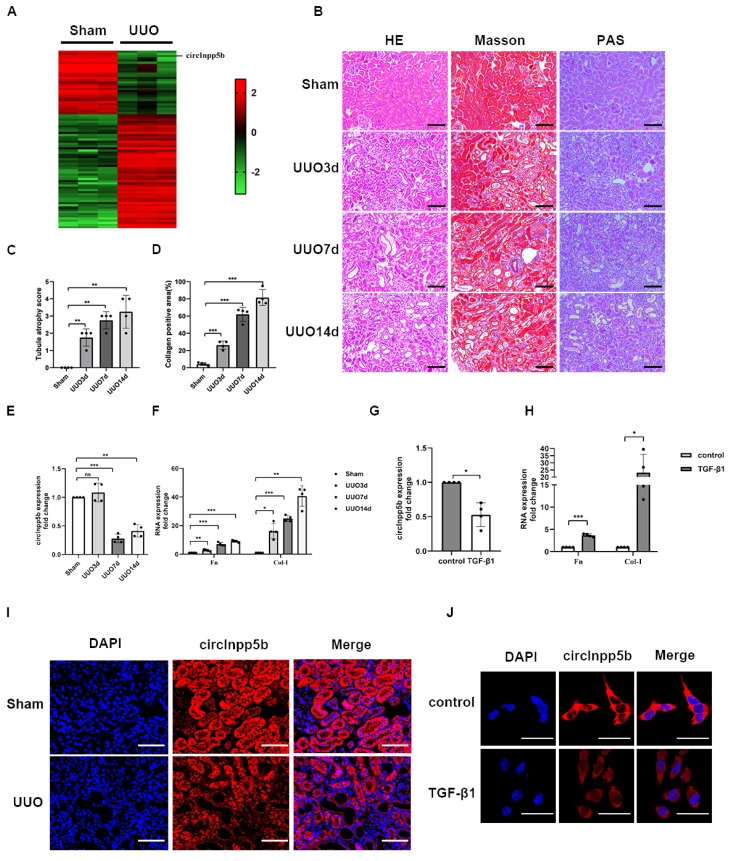
CircInpp5b is reduced in RIF models in vivo and in vitro. (**A**) High–throughput sequencing heatmap of the UUO and Sham mice (*n* = 3). (**B**) HE, Masson, and PAS staining in UUO (3, 7, and 14 days) and Sham mice (×200). Scale bar, 100 µm, (*n* = 4). (**C**) Tubule atrophy score in kidney tissues (*n* = 4). (**D**) Collagen-positive area (%) in kidney tissues (*n* = 4). (**E**–**H**) qRT-PCR confirmed the RNA levels of circInpp5b, Fn, and Col-I in RIF models (*n* = 4). (**I**,**J**) FISH assaying showed the localization of circInpp5b in mouse kidney tissues (Scale bar, 100 µm) and BUMPT cells (Scale bar, 50 µm). (*n* = 4). * *p* < 0.05, ** *p* < 0.01, *** *p* < 0.001 vs. Sham or control group.

To identify potential candidates that participated in renal interstitial fibrosis, we performed high-throughput sequencing of UUO and Sham mouse kidney tissues and screened the differentially expressed circRNAs according to standards (|log2(fc)|> 2, *p* < 0.01). The results showed that compared with the Sham group, 37 circRNAs were down-regulated in the UUO mice, while 52 were up-regulated. Furthermore, we found that compared with the Sham group, a novel circRNA, named circInpp5b, was significantly reduced in the UUO group (Figure 1A). At present, there is no relevant study on circInpp5b. Therefore, our research focused on elucidating the role underlying circInpp5b in RIF. Firstly, we harvested kidney tissues from UUO (3, 7, and 14 days) and Sham mice. HE, Masson, and PAS staining showed that compared with the Sham group, epithelial brush borders were absent, tubules were atrophied or dilated, and collagen was deposited in the renal interstitium of the UUO mice (Figure 1B–D). Then, qRT-PCR proved that circInpp5b was decreased in a time-dependent manner in the UUO mice (Figure 1E,F) and also reduced in TGF-β1-induced BUMPT cells (Figure 1G,H). Furthermore, the FISH assay confirmed that circInpp5b was mainly expressed in the renal tubular epithelial cells of mouse kidney tissues and cytoplasm of BUMPT cells (Figure 1I,J).

### 3.2. Overexpression of circInpp5b Ameliorates Renal Interstitial Fibrosis

**Figure 2:**
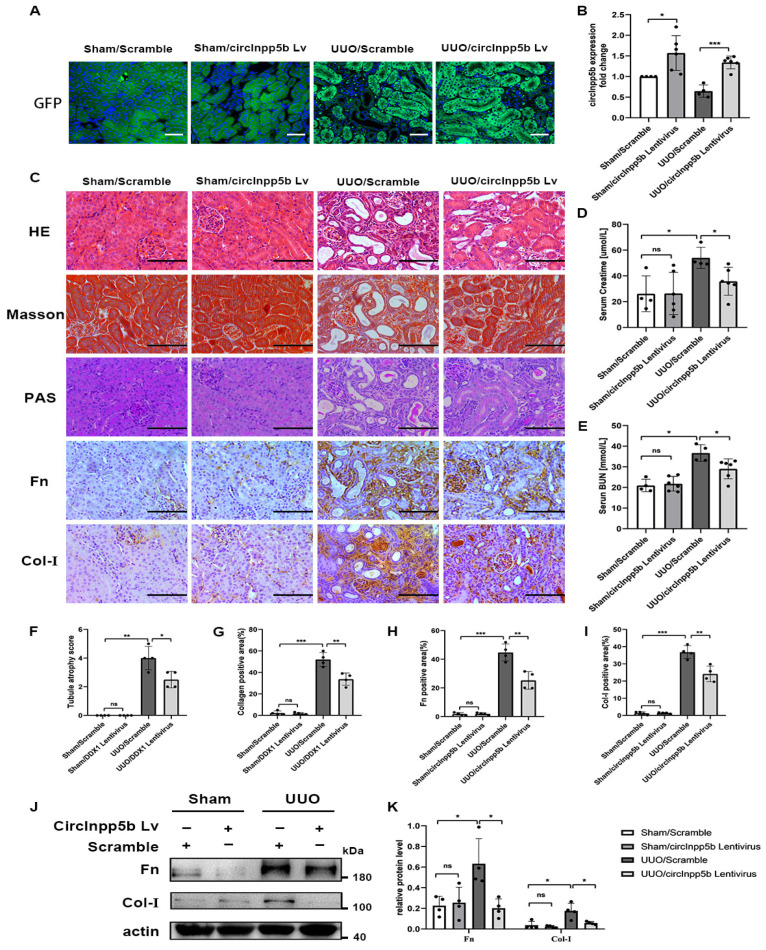
Overexpression of circInpp5b ameliorates RIF in UUO mice. (**A**) GFP fluorescence in mouse kidney tissues (×400). Scale bar, 50 µm. (**B**) qRT-PCR confirmed the expression level of circInpp5b in mouse kidney tissues. (**C**) HE, Masson, PAS, and IHC staining detected morphological changes and the ECM-related protein levels in mouse kidney tissues (×400). Scale bar, 100 µm. (**D**,**E**) Relative levels of creatinine and urea nitrogen in mice. (**F**) Tubule atrophy score in kidney tissues (*n* = 4). (**G**–**I**) Collagen-, Fn-, and Col-I-positive area (%) in kidney tissues (*n* = 4). (**J**,**K**) Western Blot and densitometry analysis detected the protein levels of Fn and Col-I in mouse kidney tissues (*n* = 4). *n* (Sham Scramble and UUO Scramble) = 4; *n* (Sham Lentivirus and UUO Lentivirus) = 6. * *p* < 0.05, ** *p* < 0.01, *** *p* < 0.001 vs. Sham/Scramble or UUO/Scramble. Original Western Blot images are available in Supplementary Materials.

To study whether circInpp5b regulates renal interstitial fibrosis, we overexpressed circInpp5b in RIF models. Firstly, in vivo, overexpression of circInpp5b in RIF animal models were constructed, and the GFP fluorescence and qRT-PCR confirmed the successful overexpression of circInpp5b (Figure 2A,B). HE, Masson, and PAS staining showed that the overexpression of circInpp5b restrained RIF in the UUO mice compared with the UUO/Scramble group (Figure 2C,F,G). Moreover, IHC staining suggested that the overexpression of circInpp5b down-regulated the protein levels of fibronectin (Fn) and collagen I (Col-I) in the renal interstitium of the UUO mice (Figure 2C,H,I). The results regarding creatinine and urea nitrogen also showed that the overexpression of circInpp5b improved renal function in the UUO mice (Figure 2D,E). Our Western Blot detected that the overexpression of circInpp5b down-regulated the protein levels of Fn and Col-I in the UUO mice (Figure 2J,K).

**Figure 3:**
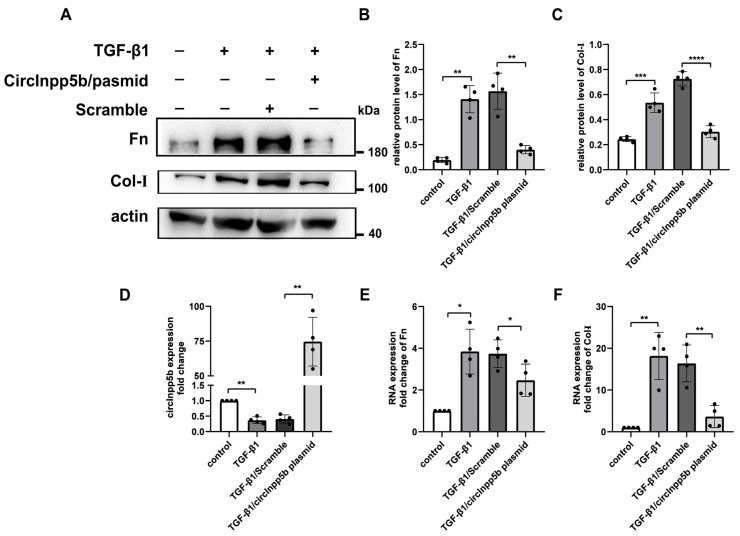
Overexpression of circInpp5b inhibits the deposition of ECM in renal tubular cells stimulated with TGF-β1. (**A**–**C**) Western Blot and densitometry analysis detected the ECM-related protein levels in renal tubular cells (*n* = 4). (**D**–**F**) qRT-PCR confirmed the RNA levels of circInpp5b, Fn, and Col-I in renal tubular cells (*n* = 4). * *p* < 0.05, ** *p* < 0.01, *** *p* < 0.001, **** *p* < 0.0001 vs. control or TGF-β1/Scramble. Original Western Blot images are available in Supplementary Materials.

In vitro, TGF-β1-induced BUMPT cells were transfected with circInpp5b overexpression plasmid, and our qRT-PCR assay confirmed that the expression of circInpp5b was up-regulated in the TGF-β1/circInpp5b plasmid group compared with the TGF-β1/Scramble group (Figure 3D). Western Blot and qRT-PCR revealed that the overexpression of circInpp5b down-regulated the protein and mRNA levels of Fn and Col-I in renal tubular epithelial cells stimulated with TGF-β1 (Figure 3A–C,E,F).

### 3.3. Knockdown of circInpp5b Promotes ECM-Related Protein Deposition In Vitro

**Figure 4:**
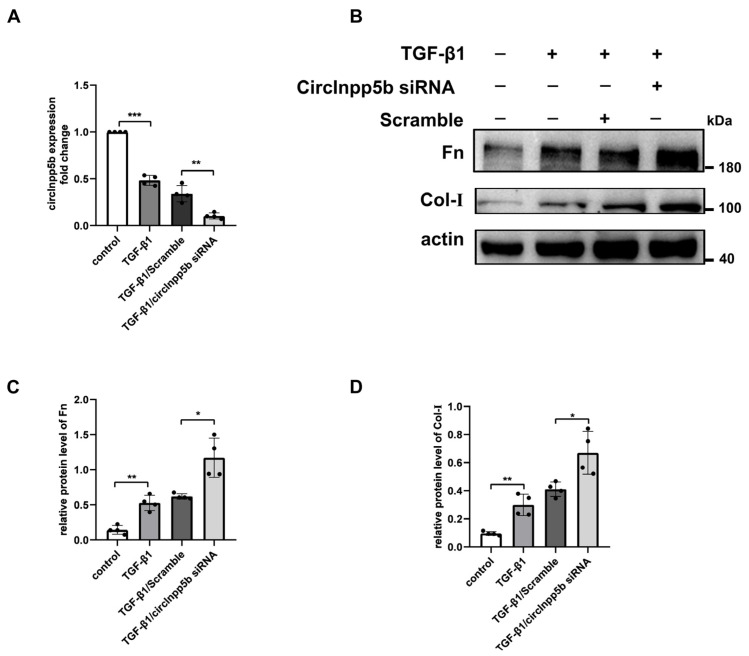
Knockdown of circInpp5b promotes the deposition of ECM in renal tubular cells stimulated with TGF-β1. (**A**) qRT-PCR confirmed the RNA level of circInpp5b in renal tubular cells (*n* = 4). (**B**–**D**) Western Blot and densitometry analysis detected the ECM-related protein levels in renal tubular cells (*n* = 4). * *p* < 0.05, ** *p* < 0.01, *** *p* < 0.001 vs. control or TGF-β1/Scramble. Original Western Blot images are available in Supplementary Materials.

In order to investigate whether the knockdown of circInpp5b relates to worse renal injury. In vitro, we transfected circInpp5b siRNA into TGF-β1-induced BUMPT cells, and the qRT-PCR assay confirmed that the expression of circInpp5b was down-regulated in the TGF-β1/circInpp5b siRNA group compared with the TGF-β1/Scramble group (Figure 4A). Our Western Blot revealed that compared with the TGF-β1/Scramble group, the expression levels of the ECM-related proteins Fn and Col-I were up-regulated in the TGF-β1/circInpp5b siRNA group (Figure 4B,C).

### 3.4. Overexpression of circInpp5b Down-Regulates the Expression of DDX1 Protein in RIF Models

**Figure 5:**
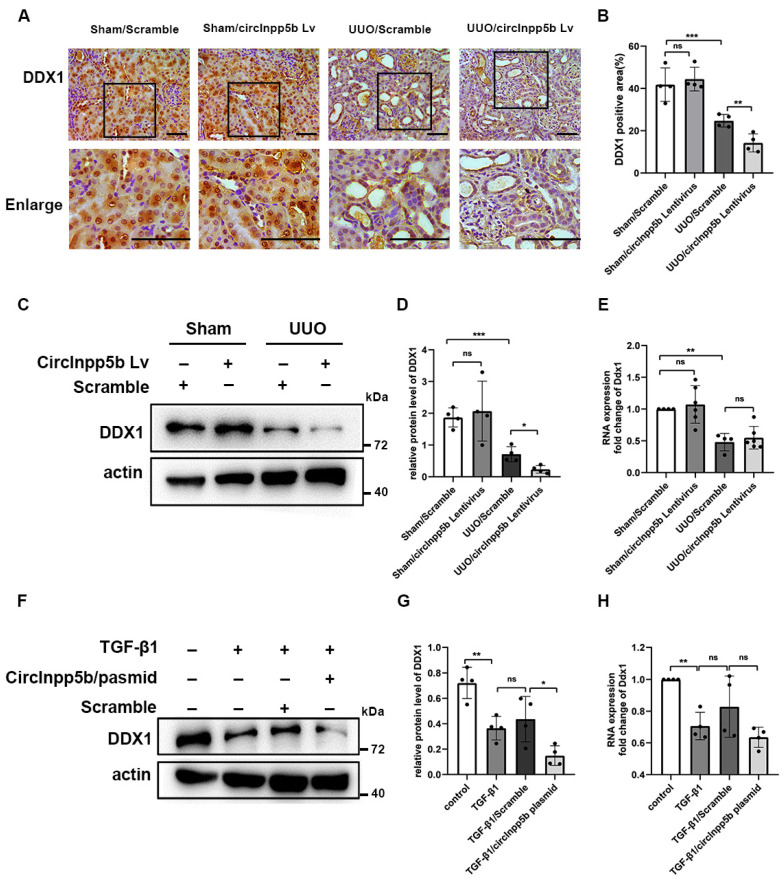
Overexpression of circInpp5b decreases DDX1 protein expression levels in RIF models. (**A**) IHC staining showed the expression level of the DDX1 protein in mouse kidney tissues (×400). Scale bar, 50 µm. *n* (Sham Scramble and UUO Scramble) = 4; *n* (Sham Lentivirus and UUO Lentivirus) = 6. (**B**) DDX1 protein-positive area (%) in kidney tissues (*n* = 4). (**C**,**D**) Western Blot and densitometry analysis detected the protein level of DDX1 in mouse kidney tissues. *n* = 4. (**F**,**G**) Western Blot and densitometry analysis detected the protein level of DDX1 in renal tubular cells (*n* = 4). (**E**,**H**) qRT-PCR confirmed the mRNA level of Ddx1 in renal tubular cells (*n* = 4). * *p* < 0.05, ** *p* < 0.01, *** *p* < 0.001 vs. control or TGF-β1/Scramble or Sham//Scramble or UUO//Scramble. Original Western Blot images are available in Supplementary Materials.

Studies demonstrated that circRNAs could interact with proteins and regulate their expression [8]. To investigate whether circInpp5b interacted with proteins to exert its function, we searched the database to predict interactions between RNAs and proteins (http://pridb.gdcb.iastate.edu/RPISeq/, accessed on 8 February 2024) and found that circInpp5b might bind to the DDX1 protein (interaction probabilities: 0.8 > 0.5 were considered positive). Thus, after overexpressing circInpp5b in RIF models, we detected the protein level of DDX1. In mouse kidney tissues, IHC staining and Western Blotting revealed that the overexpression of circInpp5b reduced the levels of the DDX1 protein in the UUO mice (Figure 5A–D). The Western Blot results suggested that the overexpression of circInpp5b down-regulated the protein level of DDX1 in the TGF-β1-treated BUMPT cells (Figure 5F,G). However, qRT-PCR showed that the overexpression of circInpp5b had no effect on the mRNA level of Ddx1 (Figure 5E,H).

### 3.5. DDX1 Protein Is Down-Regulated in RIF Models

**Figure 6:**
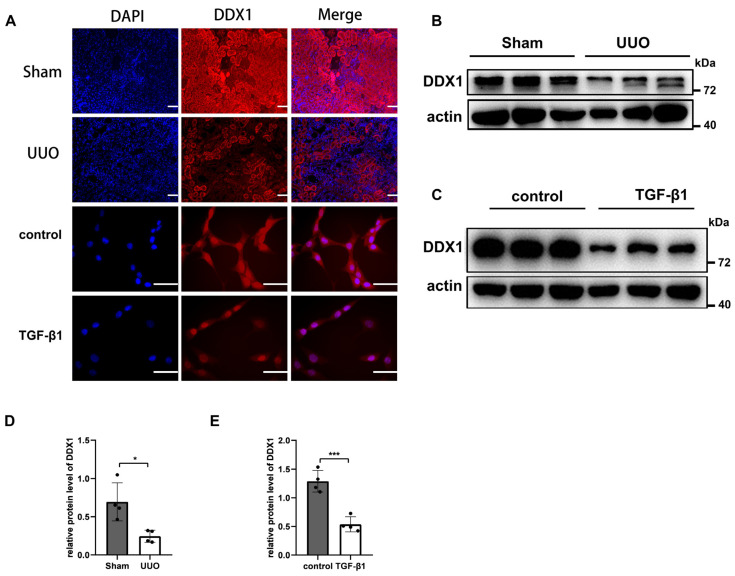
Profibrotic changes induce DDX1 down-regulation. (**A**) Immunofluorescence showed the localization and expression of DDX1 protein in mouse kidney tissues and BUMPT cells. Scale bar, 50 µm, (*n* = 4). (**B**–**E**) Western Blot and densitometry analysis detected the protein level of DDX1 in mouse kidney tissues and BUMPT cells (*n* = 4). * *p* < 0.05, *** *p* < 0.001 vs. control or Sham. Original Western Blot images are available in Supplementary Materials.

In the present study, we detected the localization and expression of DDX1 in RIF models. Immunofluorescence confirmed that DDX1 was mainly expressed in renal tubular cells (Figure 6A). Western Blotting revealed a significant decrease in the protein level of DDX1 in UUO mice and renal tubular cells stimulated with TGF-β1 (Figure 6B–E).

### 3.6. DDX1 Promotes Renal Interstitial Fibrosis In Vivo and In Vitro

**Figure 7:**
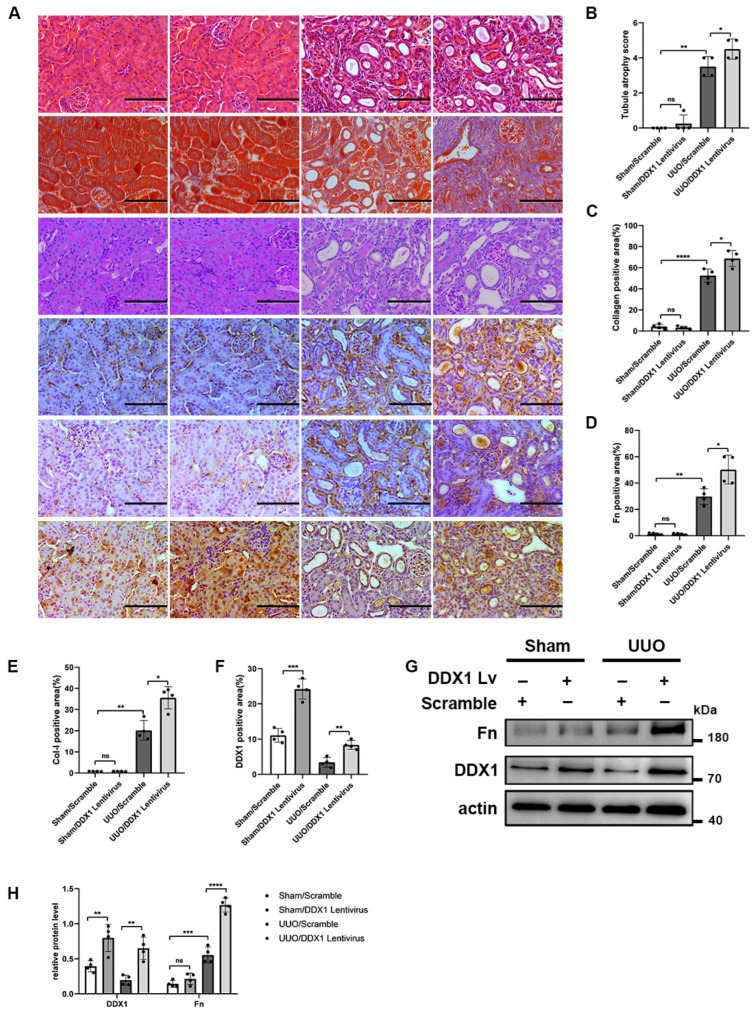
Overexpression of DDX1 aggravates RIF in UUO mice. (**A**) HE, Masson, PAS, and IHC staining detected morphological changes and the ECM-related protein levels in mouse kidney tissues (×400). Scale bar, 100 µm. (**B**) Tubule atrophy score in kidney tissues. (**C**–**F**) Collagen-, Fn-, Col-I-, and DDX1protein-positive area (%) in kidney tissues. (**G**,**H**) Western Blot and densitometry analysis detected the protein levels of Fn and DDX1 in mouse kidney tissues. *n* = 4. * *p* < 0.05, ** *p* < 0.01, *** *p* < 0.001, **** *p* < 0.0001 vs. Sham/Scramble or UUO/Scramble. Original Western Blot images are available in Supplementary Materials.

To study whether DDX1 regulates RIF in vivo, we overexpressed DDX1 in RIF models. HE, Masson, and PAS staining showed that the overexpression of DDX1 aggravated RIF in the UUO mice compared with the UUO/Scramble group (Figure 7A–C). Moreover, IHC staining suggested that the overexpression of DDX1 up-regulated the protein levels of Fn and Col-I in the renal interstitium of the UUO mice (Figure 7A,D–F). Western Blotting detected that the overexpression of DDX1 up-regulated the protein levels of Fn in the UUO mice (Figure 7G,H).

**Figure 8:**
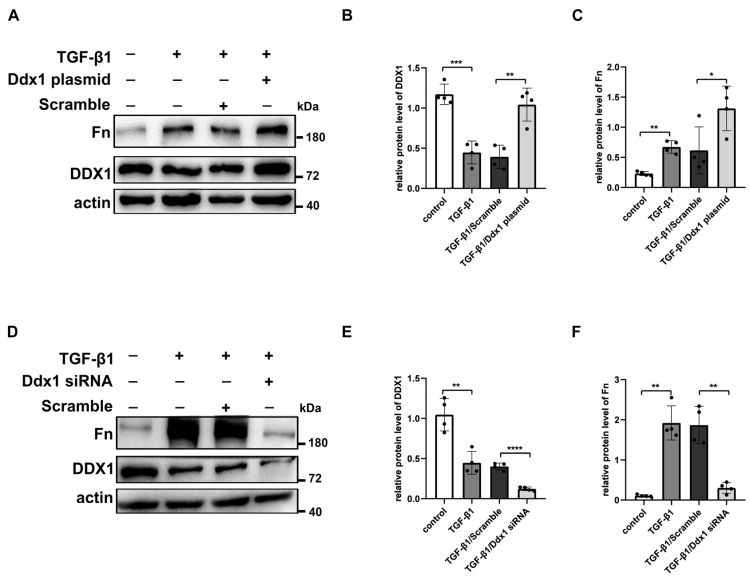
DDX1 aggravates RIF in TGF-β1-treated BUMPT cells. (**A**–**C**) Western Blot and densitometry analysis detected the protein levels of Fn and DDX1 in TGF-β1-treated BUMPT cells with Ddx1 overexpression plasmid. (**D**–**F**) Western Blot and densitometry analysis detected the protein levels of Fn and DDX1 in TGF-β1-treated BUMPT cells with Ddx1 siRNA. *n* = 4. * *p* < 0.05, ** *p* < 0.01, *** *p* < 0.001, **** *p* < 0.0001vs control or TGF-β1/Scramble. Original Western Blot images are available in Supplementary Materials.

Furthermore, renal tubular cells stimulated with TGF-β1 were transfected with DDX1 overexpression plasmid. The Western Blot results suggested that the overexpression of DDX1 up-regulated the protein level of Fn in renal tubular cells stimulated with TGF-β1 (Figure 8A–C).

In order to investigate whether the knockdown of DDX1 alleviates renal injury, in vitro, we transfected DDX1 siRNA into TGF-β1-induced BUMPT cells. Western Blotting revealed that compared to TGF-β1/Scramble group, the expression of DDX1 was decreased and the expression of ECM-related proteins Fn and Col-I were down-regulated in the TGF-β1/DDX1 siRNA group (Figure 8D–F).

### 3.7. CircInpp5b Binds to DDX1 Protein and Promotes Its Lysosomal Degradation

**Figure 9:**
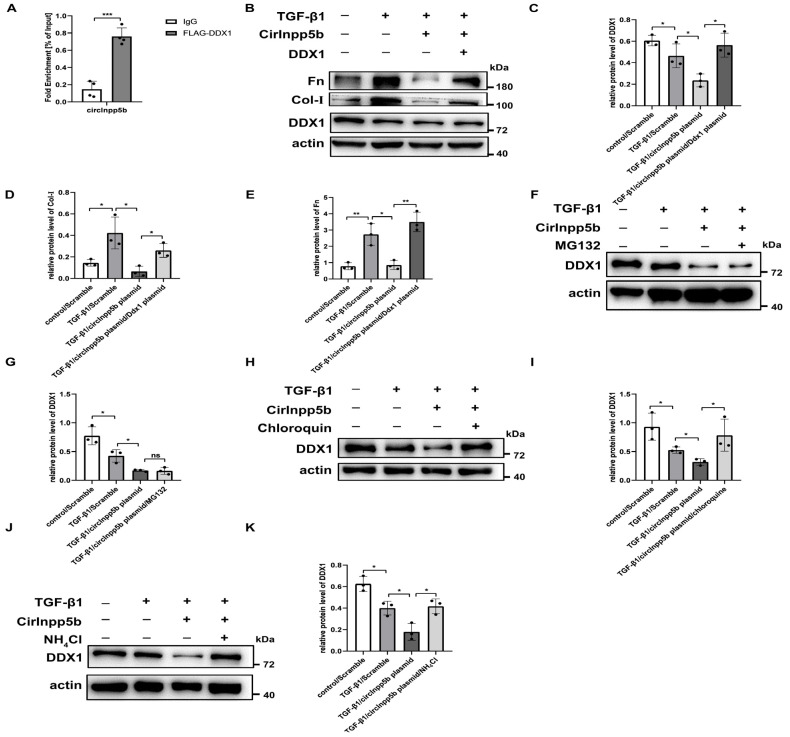
CircInpp5b interacts with DDX1 protein and promotes its lysosomal degradation. (**A**) RIP assay confirmed the binding between circInpp5b and DDX1 in 293T cells (*n* = 4). (**B**–**E**) Western Blot and densitometry analysis detected the protein levels of DDX1, Fn, and Col-I in renal tubular cells (*n* = 3). (**F**–**K**) Western Blot and densitometry analysis detected the protein level of DDX1 in renal tubular cells treated with MG132, chloroquine, and NH_4_Cl (*n* = 3). * *p* < 0.05, ** *p* < 0.01, *** *p* < 0.001 vs. IgG or control or TGF-β1/Scramble or TGF-β1/circInpp5b plasmid. Original Western Blot images are available in Supplementary Materials.

**Figure 10:**
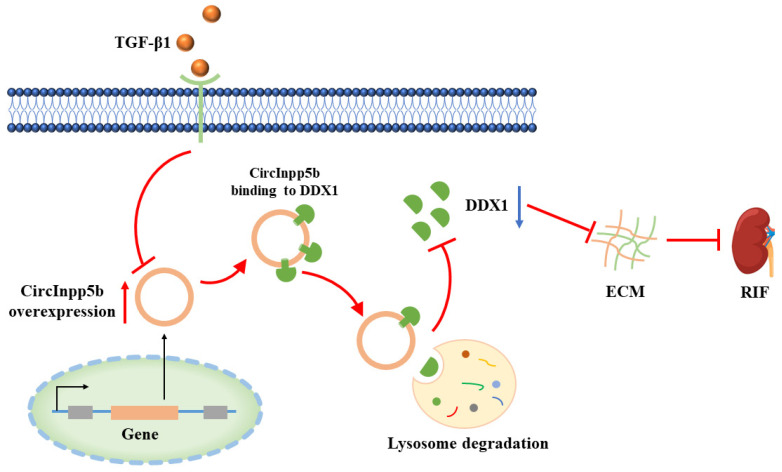
In TGF-β1-stimulated renal tubular cells, the expression of circInpp5b is reduced. The overexpression of circInpp5b promotes its binding to the DDX1 protein, thereby promoting DDX1 protein lysosomal degradation, which causes a decrease in the expression of DDX1, finally achieving the results of inhibiting ECM deposition and RIF.

As mentioned above, in this study, we found that the overexpression of circInpp5b down-regulated the protein level of DDX1 but had no effect on its mRNA level (Figure 4). It was reported that circRNAs could interact with proteins and participate in transcription, translation, post-translational modification, etc. [8]. Based on that, we explored the regulatory mechanism underlying circInpp5b’s effect on the DDX1 protein. When 293T cells were transfected with circInpp5b and DDX1 overexpression plasmids, the RIP assay proved that circInpp5b bound to the DDX1 protein (Figure 9A). Western Blotting showed that the simultaneous overexpression of circInpp5b and DDX1 reversed the inhibitory fibrotic effect of circInpp5b overexpression alone in renal tubular cells stimulated with TGF-β1 (Figure 9B–E). In order to investigate whether circInpp5b regulated the degradation of the DDX1 protein, we applied MG132 (a ubiquitination inhibitor), chloroquine (an autophagy inhibitor), or NH_4_Cl (a lysosomal inhibitor) to TGF-β1-stimulated renal tubular cells. The Western Blot results suggested that MG132 had no effect on the protein level of DDX1 in the TGF-β1-stimulated renal tubular cells (Figure 9F,G), while the protein level of DDX1 was up-regulated by chloroquine (Figure 8H,I) and NH_4_Cl (Figure 9J,K), indicating that the overexpression of circInpp5b promoted the lysosomal degradation of the DDX1 protein (Figure 10).

## 4. Discussion

CircRNAs have gradually become a research hotspot recently. There is substantial evidence that circRNAs are involved in the pathogenesis of certain fibrotic diseases. Knocking down circRNA-15698 with small interference RNAs resulted in a significant reduction in the expression levels of fibrosis-associated proteins. Mechanistically, circRNA-15698 acted as a miR-185 sponge, elevating TGF-β1 protein levels and stimulating the production of ECM-related proteins [31]. CircActa2 promoted the expression of the α-SMA protein in vascular smooth muscle cells by sponging miR-548f-5p [32]. CircRNA_010567 acted as a sponge of miR-141 and bound to TGF-β1, regulating the expression of fibrosis-related proteins in fibroblasts [33]. Considering the commonality of the key factors and pathways in the fibrosis mechanism, including TGF-β1 and epithelial-to-mesenchymal transition-associated proteins, these findings may also provide evidence for renal fibrosis. Several studies have explored the role of circRNAs in renal fibrosis. For example, the overexpression of circHIPK in a folic acid-induced renal tubulointerstitial fibrosis model down-regulated miR-30a and up-regulated profibrotic genes such as TGF-β1, Fn, and Col-I. The results suggested that circHIPK3 acted as a miR-30a sponge, exacerbating RIF [20]; knocking down circACTR2 boosted miR-200c expression, reduced YAP levels, and reduced the levels of M2 macrophages in obstructed kidney and ameliorated UUO-induced RIF. The study showed that circular RNA ACTR2 activated YAP signaling and contributed to renal fibrosis [24]. The overexpression of circPlekha7 or knockdown of miR-493-3p suppressed TGF-β1-induced enhancements in the epithelial-to-mesenchymal transition and fibrogenesis, as well as attenuated renal fibrosis and injury in mice subjected to unilateral ureteral obstruction. This study showed that circPlekha7 targeted miR-493-3p and suppressed renal fibrosis [34]. While the role and mechanisms underlying circRNAs in RIF remain unclear, our study presents the initial finding that circInpp5b inhibits RIF. As our primary focus was on the downstream mechanism of circInpp5b, the mechanism of the down-regulation of circInpp5b is still ambiguous. A study showed that miR-671 regulated degradation of circRNA CDR1 by AGO2 [35]. Another study showed that circRNAs were degraded by RNA modification recruiting endonucleases [36]. These works provide some ideas for further research in the future. All in all, our data demonstrated that circInpp5b improved RIF.

The primary function of circRNAs is performed through their role as miRNA sponges, and the second important function is performed through the interaction with proteins. CircRNAs interact with proteins and affect the expression or function of proteins. CircARSP91 could increase the susceptibility of HCC cells to NK cell cytotoxicity by up-regulating UL16 binding protein 1 (ULBP1) expression [37]. Studies have demonstrated that circZKSCAN1 exerts its inhibitive role by competitively binding FMRP, therefore blocking the binding between FMRP and β-catenin-binding protein–cell cycle and apoptosis regulator 1 (CCAR1) mRNA and subsequently restraining the transcriptional activity of Wnt signaling [38]. FECR1 bound to the FLI1 promoter in cis and recruits TET1, a demethylase that was actively involved in DNA demethylation. FECR1 also down-regulated DNMT1, a methyltransferase that is essential for the maintenance of DNA methylation [39]. Similar to these studies, our work showed that circInpp5b could bind to the DDX1 protein. Interestingly, in this study, we confirmed that the overexpression of circInpp5b down-regulated the protein level of DDX1 but did not affect its mRNA level. Therefore, we speculated that post-translational modifications of proteins might be involved in our study. It is now generally accepted that the lysosomal system and ubiquitin-proteosome system are the two principal pathways for protein degradation, including the degradation of cytoplasmic proteins and damage to organelles by lysosomes through autophagy, i.e., the autophagy–lysosomal pathway [40,41]. Our results further show that in BUMPT cells, MG132 (a ubiquitination inhibitor) had no effect on the protein level of DDX1, while chloroquine (an autophagy inhibitor) and NH_4_Cl (a lysosomal inhibitor) up-regulated the expression of the DDX1 protein, indicating that circInpp5b promoted the degradation of the DDX1 protein through the lysosomal pathway. In agreement with our findings, one study found that circFoxo3 bound to the p53 protein and promoted the ubiquitination of the p53 protein [42]. Similarly, another study found that circNfix bound to the YBX1 protein and promoted its ubiquitination process [43]. Thus, there are few studies on circRNAs regulating protein degradation pathways. Moreover, there is a lack of research on the lysosomal degradation pathway. Therefore, the results of our study have a certain novelty. Taken together, the findings of our study demonstrate that circInpp5b bound to the DDX1 protein and promoted the degradation of it through the lysosomal pathway.

DDX1 is involved in RNA metabolism, virus replication, and the development of tumors [25]. Studies showed that DDX1 accelerated the occurrence of tumors, including neuroblastoma, colorectal and breast cancer, etc. [44,45]. A study showed that the depletion of endogenous DDX1 by specific small interfering RNAs significantly reduced NF-κB-dependent transcription. Taken together, the results suggest that DDX1 may play an important role in NF-κB-mediated transactivation [46] and that the TGF-β1/NF-κB pathway was involved in RIF [2], which provides new ideas for mechanistic research regarding DDX1 in RIF. Our study provides evidence that DDX1 promotes RIF. However, the precise mechanism underlying DDX1’s role in RIF remains to be investigated. In brief, DDX1 is a factor promoting RIF.

To sum up, our study elucidates the beneficial role of circInpp5b in RIF. Moreover, the protective effect of circInpp5b was related to the lysosomal degradation of the DDX1 protein, bringing novel insights into the mechanisms of circRNAs in RIF. We believe that circInpp5b and the DDX1 protein can be clinical therapeutic targets for CKD. Nevertheless, there are some limitations to our study. The role and mechanism of circInpp5b could be verified in different cell models, and the mechanism underlying DDX1’s role in RIF needs to be investigated in the future.

## Figures and Tables

**Table 1:** Primer sequences for qRT-PCR.

Gene	Forward	Reverse
CircInpp5b	CTGCCAGGACCATCTTTGAT	AGGGAACTGCATTCGAGAAC
Ddx1	CTCCGAAATGGGTGTTATGCC	ATGGGATAGATTCTGCCTGGAT
Fn	GTGGCTGCCTTCAACTTCTC	TTGCAAACCTTCAATGGTCA
Col-I	ATGTCGCTATCCAGCTGACC	CCTTCTTGAGGTTGCCAGTC
β-actin	TCACCCACACTGTGCCCATCATCGA	CAGCGGAACCGCTCATTGCCAATGG

## Data Availability

Data are contained within the article.

## Supplementary Materials

The following supporting information can be downloaded at: https://www.mdpi.com/article/10.3390/biom14060613/s1, Supplementary File: Original Western Blot images.
