# Supplementary material for: CircInpp5b Ameliorates Renal Interstitial Fibrosis by Promoting the Lysosomal Degradation of DDX1
Source: Biomolecules. 2024 May 23;14(6):613. doi: 10.3390/biom14060613 (PMC11201918; doi:10.3390/biom14060613)

Original Images

Figure 2F

1

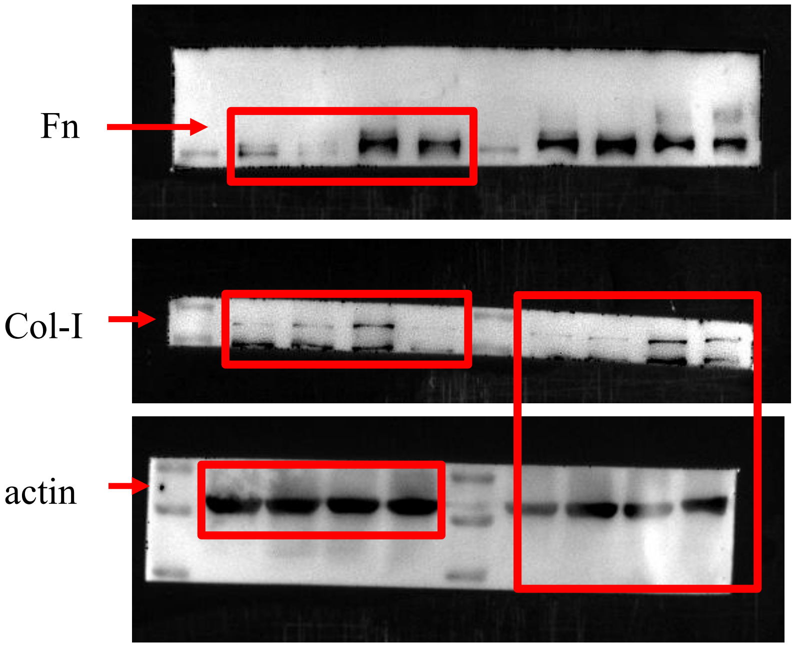

1 (Membrane combination)

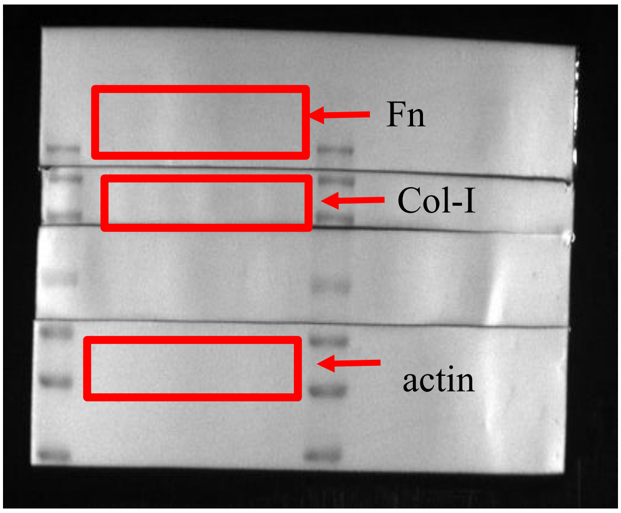

2

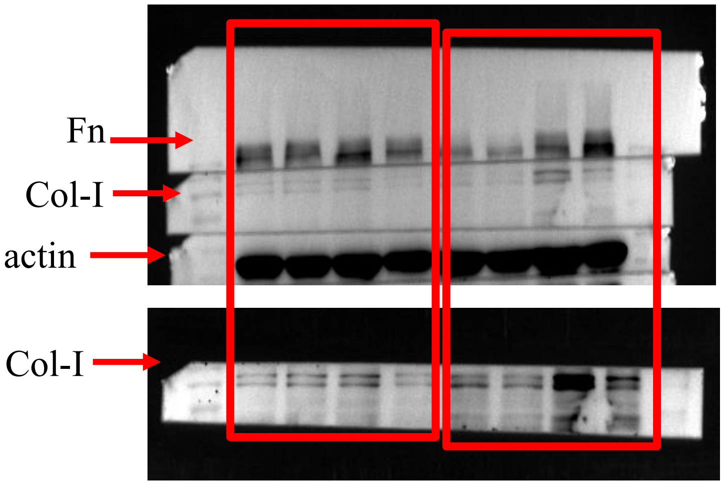

3

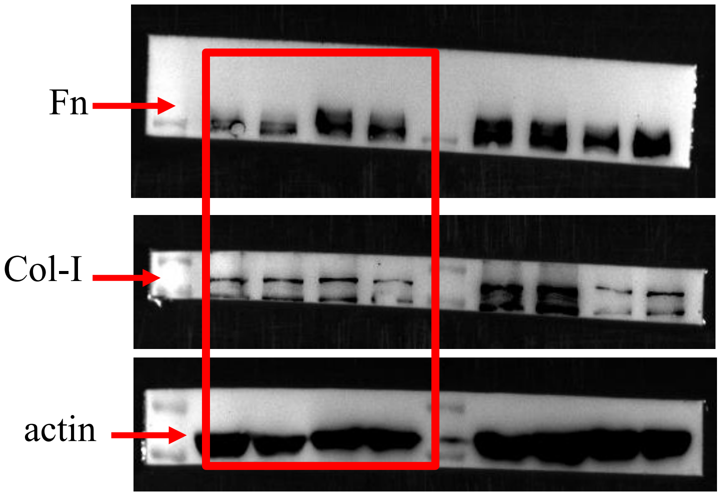

Figure 3A

1

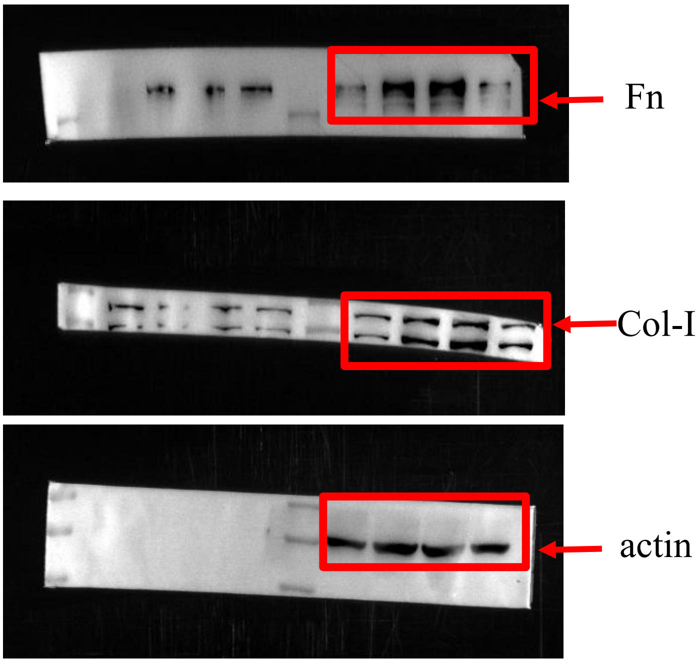

1 (Membrane combination)

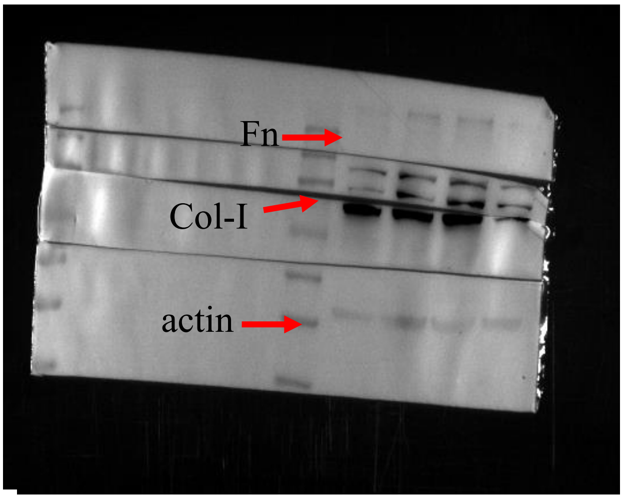

2 Fn

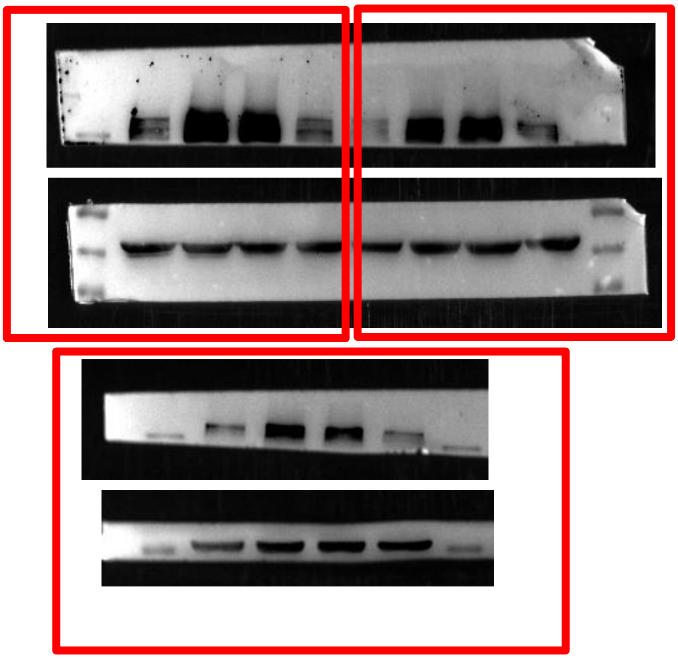

3 Col-I

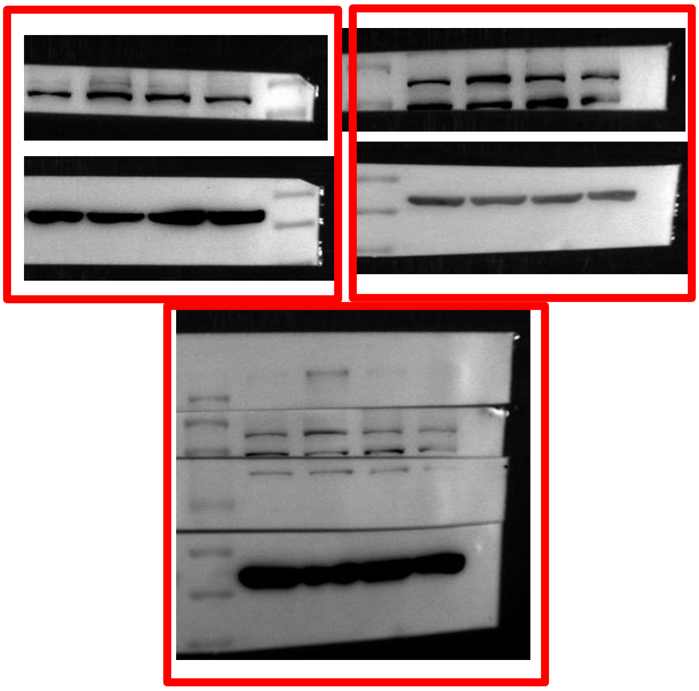

Figure 4B

1

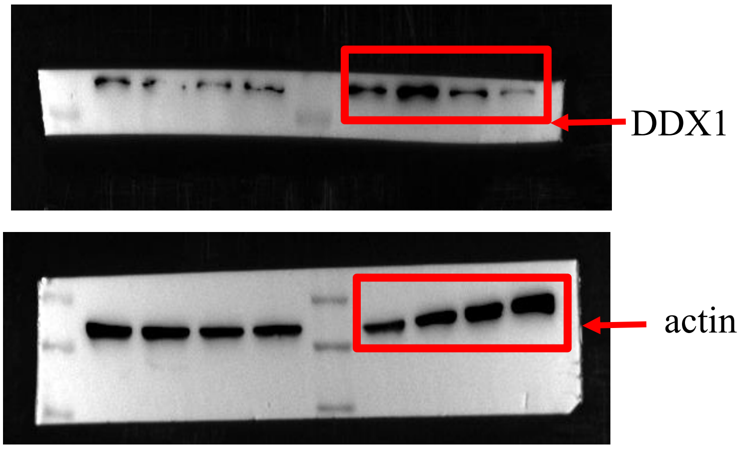

2

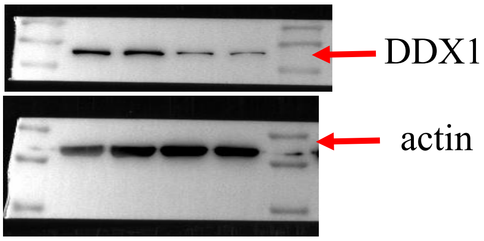

3

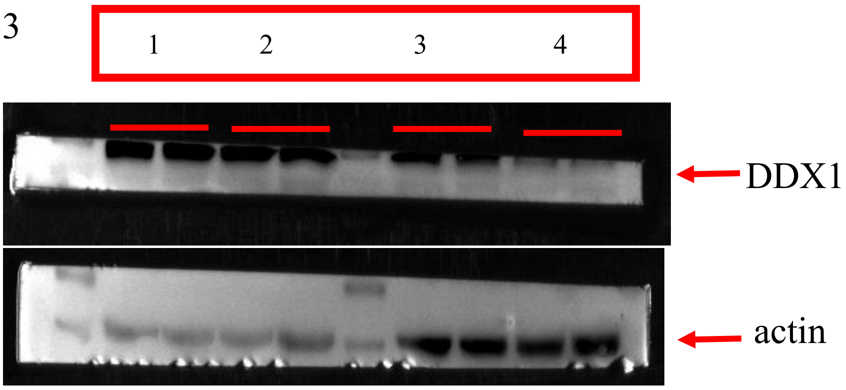

Figure 4E

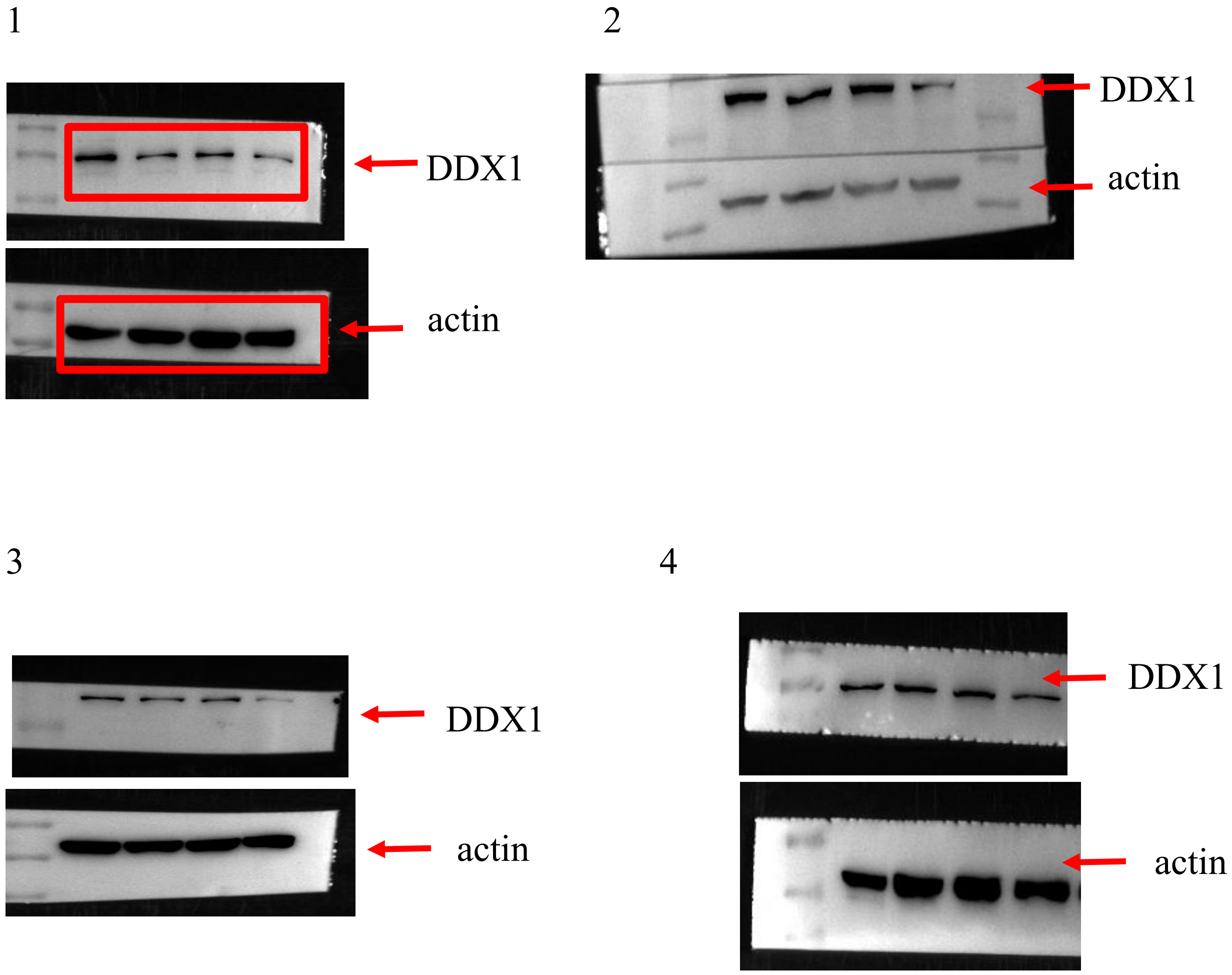

Figure 5B

1

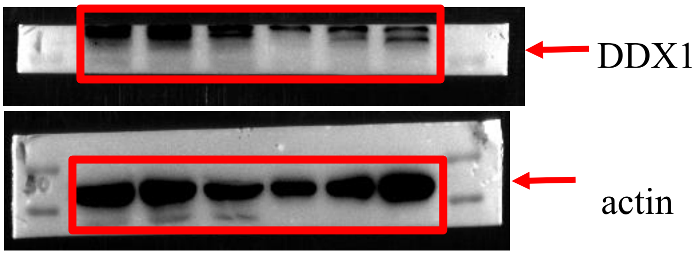

1 (Membrane combination)

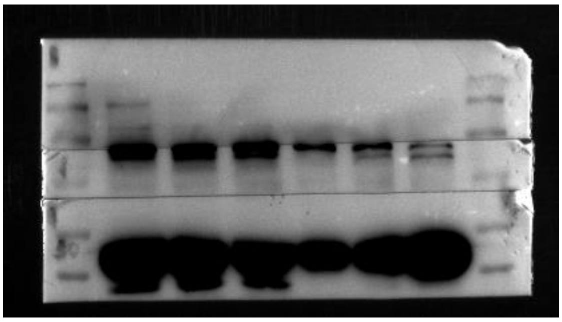

Figure 5C

1

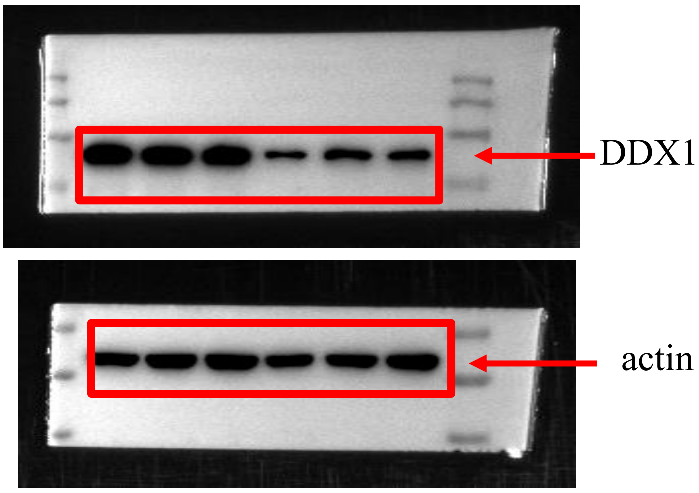

1 (Membrane combination)

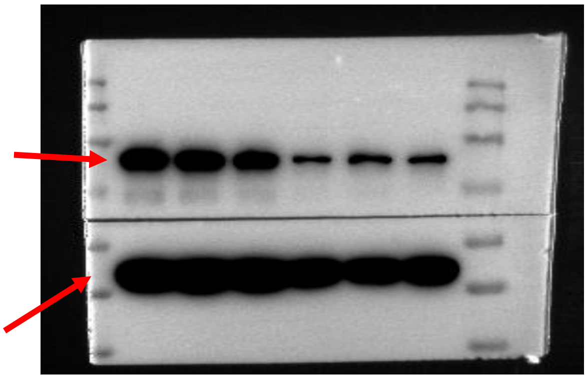

Figure 5F

1

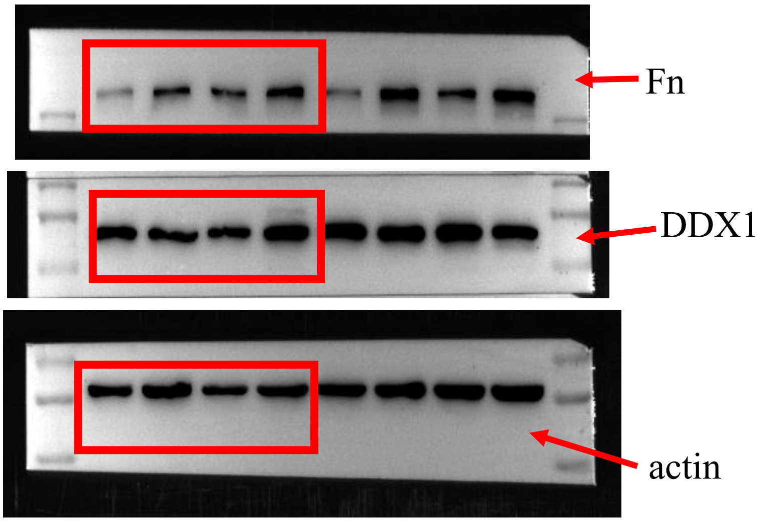

1 (Membrane combination)

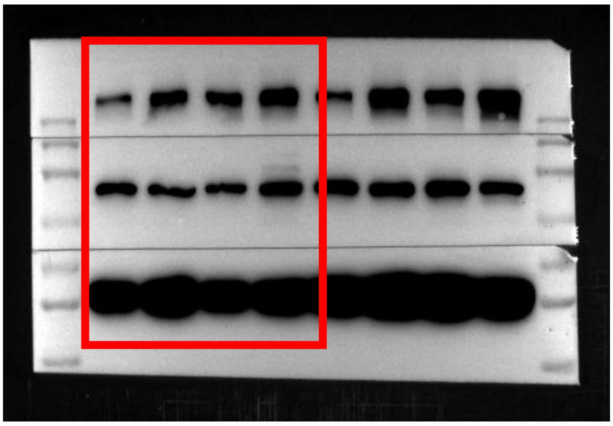

2 DDX1

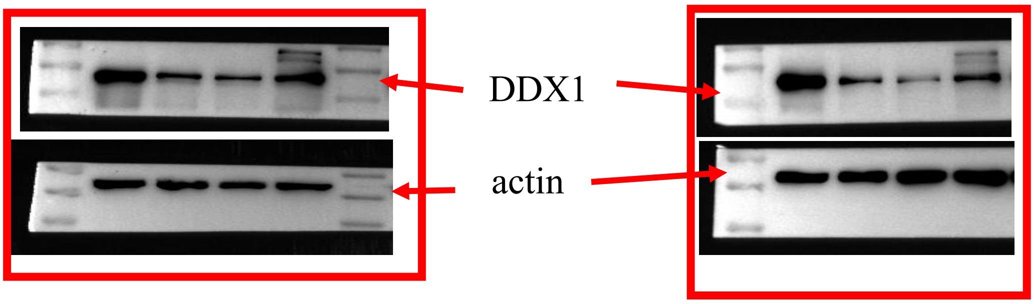

3 Fn

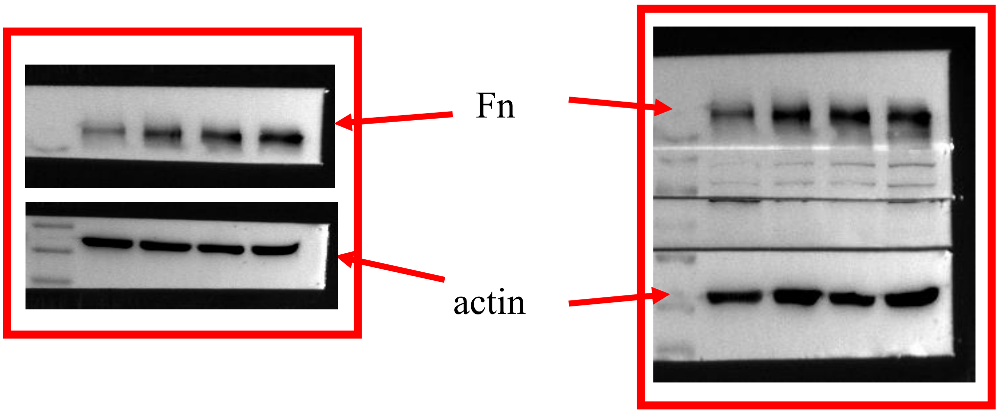

Figure 6B

1

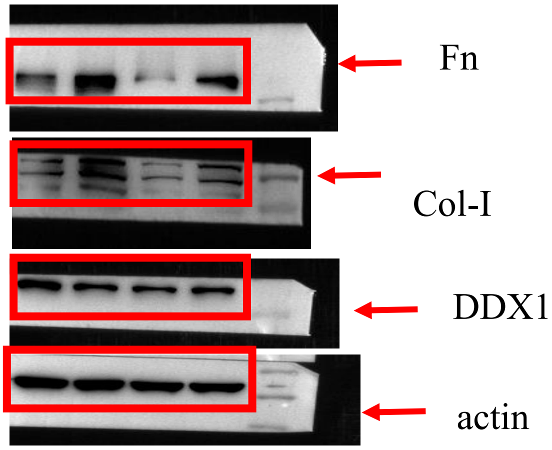

2 DDX1

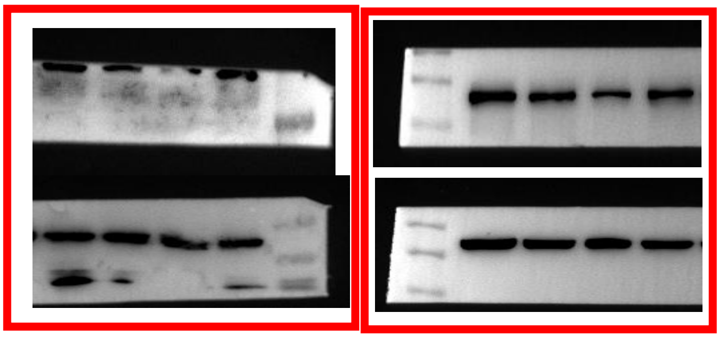

3 Fn

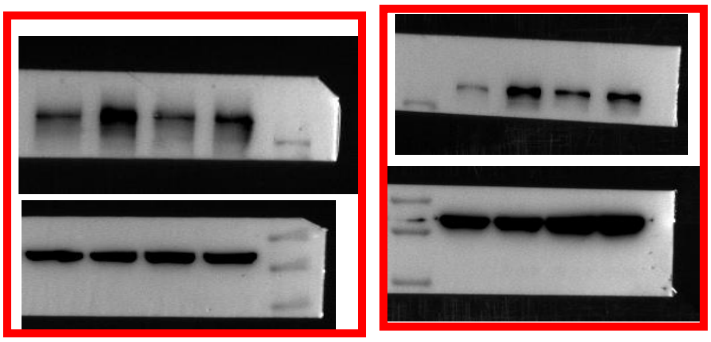

3 Col-I

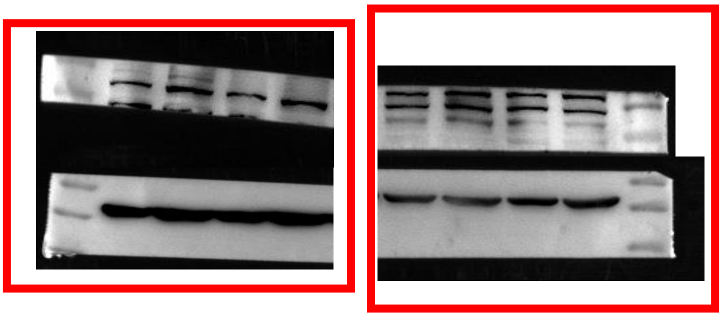

Figure 6F

1

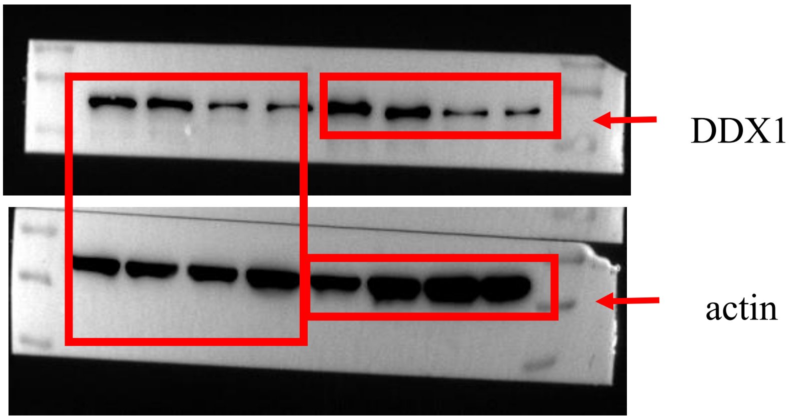

2

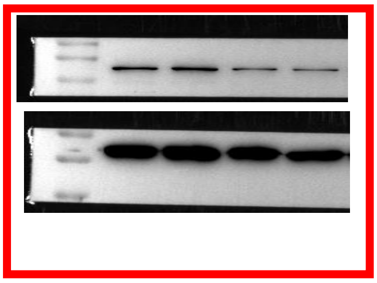

Figure 6H

1

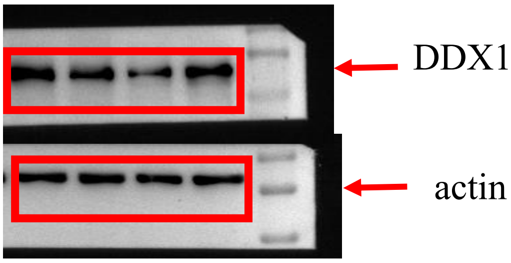

2

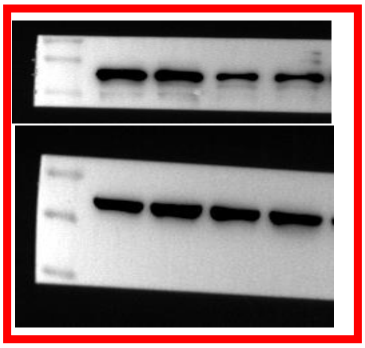

3

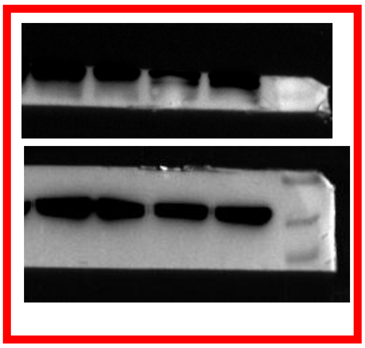

Figure 6J

1

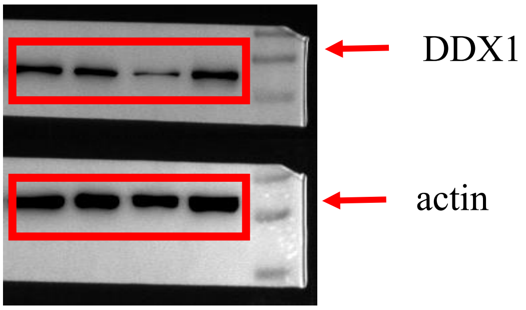

1 (Membrane combination)

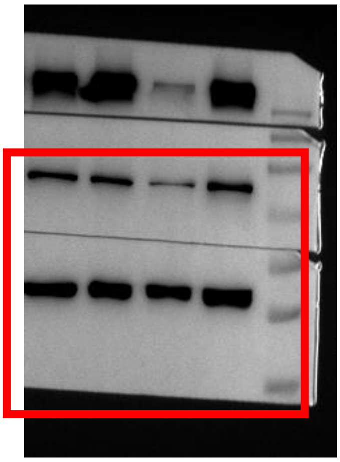

2

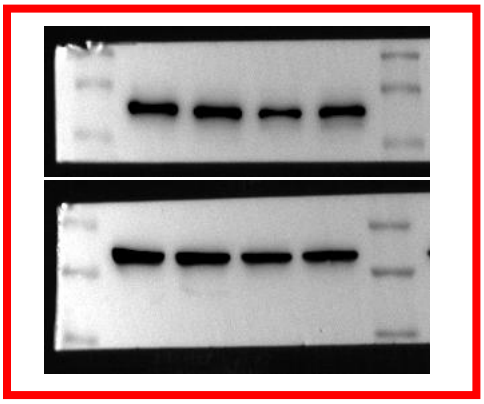

2 (Membrane combination)

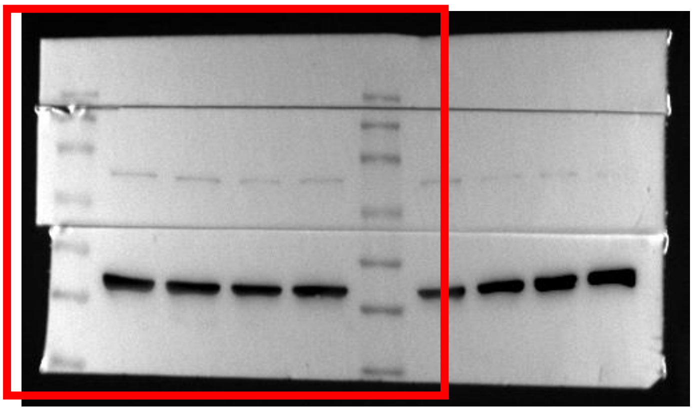

3

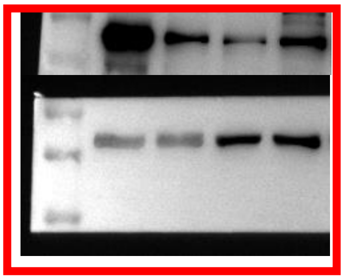

Supplement: Supplementary file 1 [file biomolecules-14-00613-s001.zip › biomolecules-2954392-supplementary.pdf]
